# Left Sided Oesophageal Lung: A Diagnostic Challenge

**DOI:** 10.1155/2013/947401

**Published:** 2013-03-06

**Authors:** Amitava Sur, Syamal Kumar Sardar, Anshuman Paria

**Affiliations:** Department of Neonatology, SSKM Hospital & IPGME&R, India

## Abstract

Bronchopulmonary foregut malformations (BPFMs) include a wide variety of malformations such as intralobar or extralobar pulmonary sequestration, foregut duplication cysts, and diverticula of the gastrointestinal or pulmonary tree (Srikanth et al., 1992). Those anomalies in which a tract between the respiratory and alimentary systems exists are termed communicating bronchopulmonary foregut malformations (CBPFMs). Most infants with CBPFMs suffer from respiratory distress, and an accurate diagnosis may be difficult to make at the patient's initial presentation. Herein we report such a case which posed a diagnostic challenge to us. This baby however survived and is doing well on a 2-year followup.

## 1. Case report

**Figure 1:**
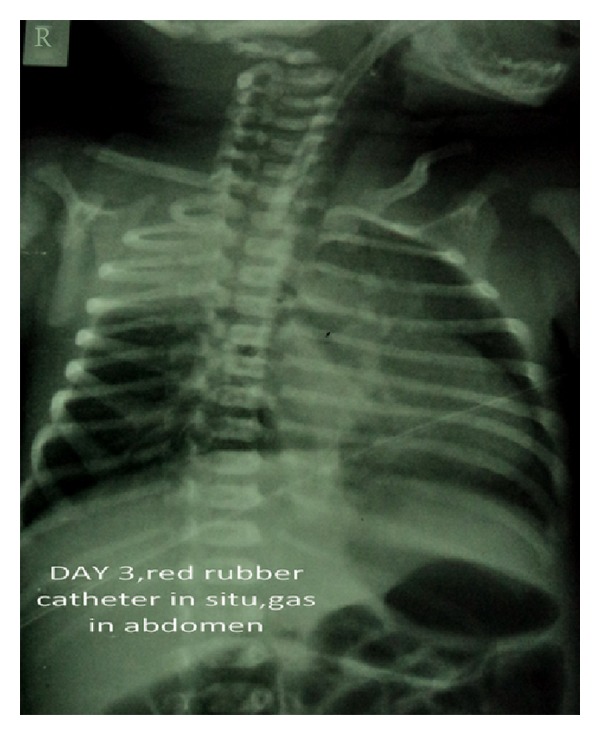
Tracheoesophageal fistula, red rubber catheter in situ.

**Figure 2:**
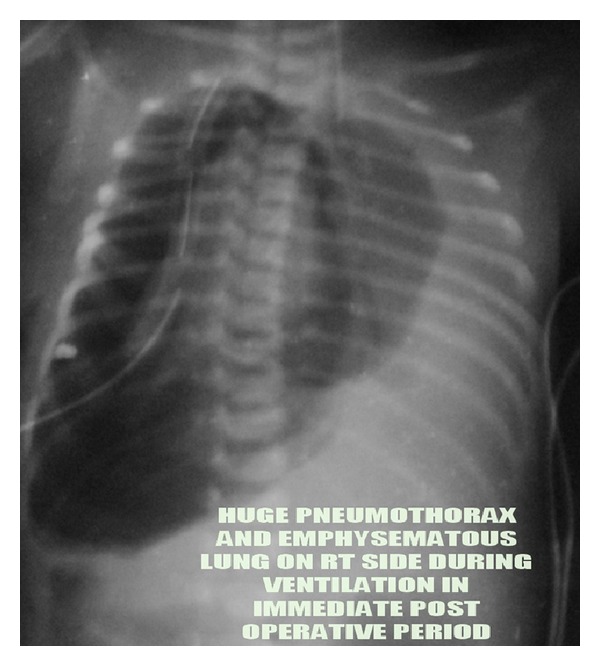
Right sided pneumothorax in the postoperative period.

**Figure 3:**
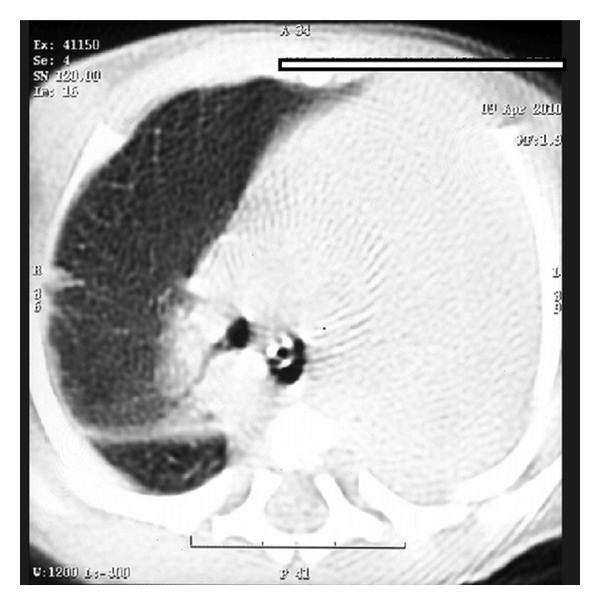
CT chest with atretic left hemithorax.

**Figure 4:**
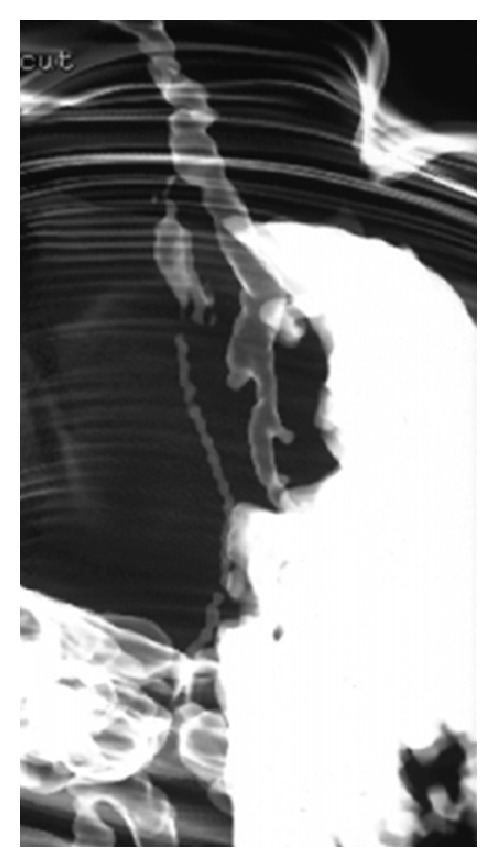
Left bronchus arising from oesophagus.

**Figure 5:**
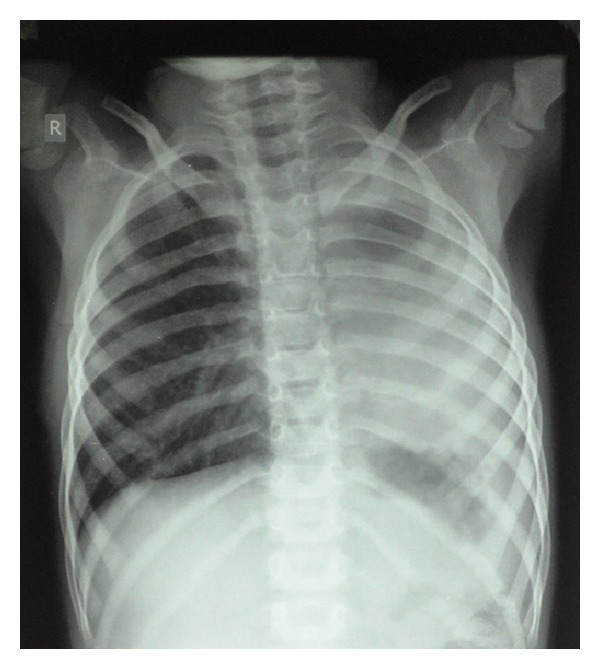
Last followup X ray at 2 years and 7 months showing left lung whiteout.

A term appropriate for gestational age female born by normal vaginal delivery presented to our Neonatal Intensive Care with complaints of excessive salivation and blood-stained frothing. No abnormality was detected antenatally. X-ray with red rubber catheter in situ and dye test revealed oesophageal atresia with tracheoesophageal fistula (TEF) (Figure 1). She underwent right sided thoracotomy with repair of TEF on day 5 of life. The baby was ventilated in the postoperative period, required persistently higher pressures, and was put on High Frequency Oscillatory ventilator. Chest X-ray (CXR) showed right sided pneumothorax with complete left sided collapse (Figure 2). Serial X-rays were performed, and though right hemithorax showed improvement after chest drain insertion, the left lung persistently collapsed. CT thorax showed that left lung was completely atretic (Figure 3). Contrast enhancement showed patent right bronchus and well-aerated right lung. It also showed rudimentary left bronchus arising from oesophagus (Figure 4). Only the proximal portion of the left main bronchus was visible. The baby was gradually weaned off ventilator and discharged from the NICU at 2 months of age. Serial followups revealed a single aerated right lung and atretic left lung both clinically and radiologically. The baby is now 2 years and 7 months old and is doing well with no respiratory assistance. Her weight and height are at the 50th percentile. Echo screening showed small muscular ventricular septal defect (VSD) 2 mm in size. The chest X-ray at the last follow-up visit shows normal right lung with left sided whiteout (Figure 5). The initial chest X-rays in the preoperative period had showed normally aerated left lung. In view of these findings we concluded that the child had the rare anomaly where the left lung originated from the distal end of the oesophagus and thus postoperatively it collapsed completely.

## 2. Discussion 

Bronchopulmonary foregut malformations are a group of rare anomalies whose exact aetiology remains obscure. One popular theory is that the close relation between the respiratory and the foregut during development accounts for this. The respiratory system develops as a ventral diverticulum arising from the foregut and is ultimately separated from the latter by the tracheoesophageal groove [1]. Abnormal formation of this groove along with differential elongation of the oesophagus and trachea may result in these anomalies. There is a strong predilection of oesophageal lungs to occur on the right side due to the proximity of the right main bronchus to the oesophagus [2]. 

The diagnosis of oesophageal lung is primarily suspected in the presence of a persistently collapsed lung, but when in association with tracheoesophageal fistula the lung may be falsely aerated as in our case initially. An oesophagogram is most helpful in delineating the second fistula. Contrast study may also be performed if a gastrostomy exists. Coexisting cardiac and other anomalies often worsen the prognosis [3]. Oesophageal atresia with TEF has been reported in about half of the cases [4]. It is the distal oesophagus to which the oesophageal lung is connected in cases of EA/TEF; that is, besides the TEF, the distal oesophagus has a bronchus arising from it with an oesophageal lung. In our patient fortunately the VSD did not pose any complication. An oesophageal lung may be mistakenly labelled as a pulmonary sequestration. The main differences between the two are as follows: first, the oesophageal lung involves the entire lung whereas the sequestration is a lobar involvement and second, the blood supply to the oesophageal lung is from the pulmonary artery but the sequestered segment has systemic supply. 

A staged reconstruction of the ligated left lung was considered by the cardiothorasic team but postponed as the baby was doing well without intervention.
